# Linkage analysis of alcoholism-related electrophysiological phenotypes: genome scans with microsatellites compared to single-nucleotide polymorphisms

**DOI:** 10.1186/1471-2156-6-S1-S156

**Published:** 2005-12-30

**Authors:** Jocelyn F Bautista, Shannon RE Quade, Antonio R Parrado, Katrina AB Goddard

**Affiliations:** 1 Department of Epidemiology and Biostatistics, Case Western Reserve University, Cleveland, OH, USA;; 2 Department of Neurology, Cleveland Clinic Foundation, Cleveland, OH, USA

## Abstract

P300 amplitude is an electrophysiological quantitative trait that is correlated with both alcoholism and smoking status. Using the Collaborative Study on the Genetics of Alcoholism data, we performed model-free linkage analysis to investigate the relationship between alcoholism, P300 amplitude, and habitual smoking. We also analyzed the effect of parent-of-origin on alcoholism, and utilized both microsatellites (MS) markers and single-nucleotide polymorphisms (SNPs). We found significant evidence of linkage for alcoholism to chromosome 10; inclusion of P300 amplitude as a covariate provided additional evidence of linkage to chromosome 12. This same region on chromosome 12 showed some evidence for a parent-of-origin effect. We found evidence of linkage for the P300 phenotype to chromosome 7 in non-smokers, and to chromosome 17 in alcoholics. The effects of alcoholism and habitual smoking on P300 amplitude appear to have separate genetic determinants. Overall, there were few differences between MS and SNP genome scans. The use of covariates and parent-of-origin effects allowed detection of linkage not seen otherwise.

## Background

A reduced amplitude of the P300 event-related potential is a heritable phenotype that correlates with alcoholism and other psychiatric disorders. Quantitative trait linkage analysis of P300 amplitude in the Collaborative Study on the Genetics of Alcoholism (COGA) subjects identified linkage to five regions on chromosomes 2, 5, 6, 13, and 17 [1,2]. Considering jointly the DSM-IV diagnosis of alcoholism and P300 amplitude with a bivariate variance component analysis, linkage was found to chromosome 4 [3]. A recent study found that reduced P300 amplitudes strongly correlate with smoking status [4]. It is unknown what effect smoking status has on the linkage results seen with alcoholism and P300 amplitude. Given the strong correlation between tobacco and alcohol dependence, it is important to determine if the linkage signal (related to P300 amplitude and alcoholism) is due to alcoholism, smoking status, or both. Parent-of-origin effects appear to have an important role in complex genetic disease and may play a role in alcoholism based on data from animal models and association studies of GABA_A _receptor alleles [5]. The objectives of this study were to detect genes underlying P300 amplitude, considering the independent and joint effects of alcoholism and habitual smoking, and to investigate the role of parent-of-origin effects in alcoholism, with the use of both microsatellite (MS) and single-nucleotide polymorphism (SNP) genome scans.

## Methods

### Data source

This study analyzed the COGA dataset released for the Genetic Analysis Workshop 14. Subject ascertainment and data collection have been previously described [6]. Genotype data was supplied for 328 MS markers and 4763 Illumina SNPs distributed across the genome. To minimize heterogeneity within the sample, we analyzed only those families that were predominantly White, non-Hispanic. We analyzed alcoholism using the COGA variable ALDX2 after recoding it as a binary trait. We coded those individuals meeting diagnostic criteria for alcoholism (ALDX2 = 5) as affected, those individuals who were exposed to alcohol and showed no symptoms of alcoholism (ALDX2 = 1) as unaffected, and the rest as missing.

As a measure of P300 amplitude, we used the COGA variable ttth4, which is measured at the posterior electrode Pz. P300 amplitude is usually recorded most reliably, shows its maximum amplitude, and is least affected by artifact in the posterior electrodes. P300 amplitude at the Pz electrode is correlated with amplitudes at other locations. The P300 amplitude variable was adjusted for sex and age using linear regression prior to linkage analysis. To evaluate the effect of smoking status on P300 amplitude, we stratified individuals into smoking and non-smoking subgroups using the COGA variable habitual smoker. To evaluate the effect of alcoholism on P300 amplitude, we stratified individuals into alcoholism and non-alcoholism subgroups, using the ALDX2-derived binary variable described above.

### Linkage analysis

Two methods of model-free linkage analyses were performed using the SIBPAL and LODPAL programs in S.A.G.E. [7]. LODPAL uses the general conditional logistic model that allows for inclusion of affected-relative-pairs (ARPs), covariates using the one-parameter modification [8], and parent-of-origin effects. Parent-of-origin effects, or genomic imprinting, refer to the differential expression of an allele depending on the sex of the parent who transmitted the allele. To incorporate a parent-of-origin effect, the conditional logistic model fits separate parameters for maternal and paternal transmission using parent-of-origin-specific identity-by-descent (IBD) values for sib pairs. Two β parameters, β_1m _and β_1p_, are estimated, as measures of the recurrence risk ratio, λ_1_, where λ_1m _= exp(β_1m_) is the recurrence risk ratio for a sib pair that shares exactly one allele IBD, the maternal allele. Testing for a parent-of-origin effect involves testing whether β_1m _= β_1p_. SIBPAL performs linear regression-based modeling of sib-pair traits as a function of marker allele IBD sharing. For quantitative traits, sib-pair regression-based linkage analysis was performed in SIBPAL using the weighted combination of squared trait difference and squared mean-corrected trait sum, further adjusted for the non-independence of sib pairs and the non-independence of squared trait sums and differences. SIBPAL models allow for binary and continuous traits, as well as inclusion of covariates and epistatic interactions. IBD sharing probabilities were estimated using GENIBD in S.A.G.E [7]. We chose *p *= 2.2 × 10^-5 ^and *p *= 7.4 × 10^-4 ^as thresholds for significant and suggestive linkage, respectively, as proposed by Lander and Kruglyak [9]. We used the permutation test available in SIBPAL to compute empirical *p*-values, for the regions with small nominal *p*-values from the asymptotic distribution. Under this test, we permuted the allele sharing (not the trait values) among the pairs (both within sibships and across sibships of the same size).

## Results

### Linkage analysis of alcoholism trait

**Figure 1:**
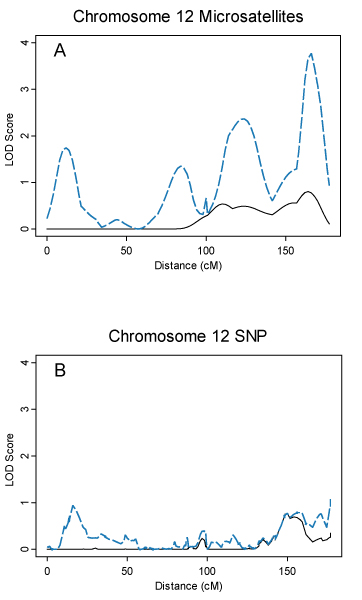
**Affected-relative-pair linkage analysis with P300 amplitude as a covariate**. Linkage results (LOD scores plotted over distance in centimorgans (cM)) are shown for MS markers (A) and SNPs (B). Solid line indicates the baseline model with ALDX2 as a binary trait, in only those individuals with P300 data available. Dashed line indicates model with P300 as a covariate. Sample sizes are 204 ARPs for MS, 200 for SNP data.

The dataset included 1,253 White, non-Hispanic individuals in 116 pedigrees, comprising 633 men and 620 women. MS marker data was available for 1,062 individuals, SNP data for 1,044 individuals. Model-free ARP linkage analysis was performed on chromosomes 1 through 22 using LODPAL. Using the binary trait for alcohol dependence, there was significant evidence for linkage to chromosome 10q using the SNP genome scan (multipoint LOD = 3.679 at 116.3 cM, *p *= 2.0 × 10^-5^). No evidence for linkage was found using the MS genome scan. Adding P300 as a covariate, suggestive evidence for linkage was seen to 12q (LOD = 3.773 at 166 cM, *p *= 0.0001; Figure 1A). The sign of the parameter estimate indicates that relative pairs with low P300 amplitudes were more likely to be linked to this locus (γ = -0.259). No evidence for linkage was seen using the SNP genome scan (Figure 1B). Repeating this analysis with habitual smoking as the defining trait, with the addition of the P300 covariate, showed no evidence for linkage to chromosome 12q (maximum multipoint LOD = 1.024 at 170 cM, *p *= 0.062) or any other region. Restricting the analyses to only those relative pairs that had both MS and SNP genome scans had no significant effect on the results above.

### Parent-of-origin linkage analysis

**Figure 2:**
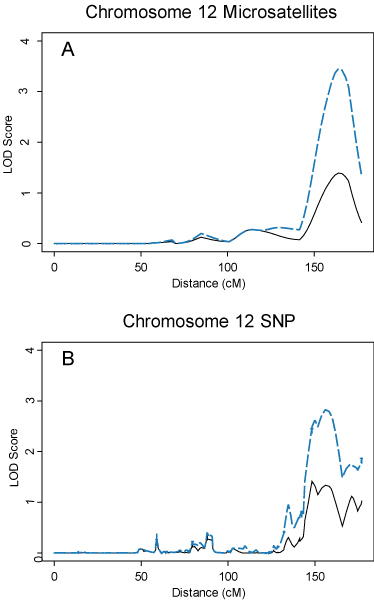
**Affected-sib-pair linkage analysis with a parent-of-origin model**. Linkage results are shown for MS markers (A) and SNPs (B). Solid line indicates the baseline model with ALDX2 as a binary trait, in affected-sib-pairs (ASPs) only. Dashed line indicates the parent-of-origin model, the test for a difference in maternal vs. paternal inheritance. Sample sizes are 321 for MS, 316 for SNP data.

Interestingly, linkage analysis under a parent-of-origin model showed some evidence for linkage to this same region of 12q (Figure 2), with both the MS (LOD diff = 2.059 at 164 cM, *p *= 0.002) and SNP (LOD diff = 1.491 at 156 cM, *p *= 0.009) genome scans. Parameter estimates indicate a maternal effect, or increased maternal transmission at this locus (β_1m _= 0.432, β_1p _= 0.0).

### Quantitative trait linkage analysis of P300 amplitude

**Figure 3:**
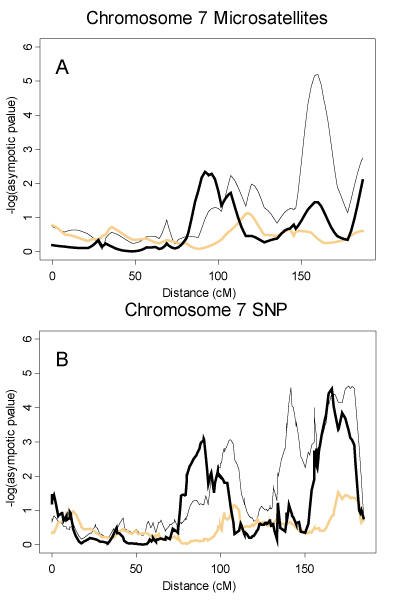
**Sib-pair QTL results of P300 amplitude, stratified by smoking status**. Linkage results are shown for MS markers (A, *N *= 735 sib-pairs) and SNPs (B, *N *= 718 sib-pairs). The negative log of the asymptotic *p*-value is plotted over distance in cM. Results are shown for the entire group (thin black line), the smoking subgroup (thick tan line), and the non-smoking subgroup (thick black line). Sample sizes in sib-pairs are 228 for MS smoking, 220 for MS non-smoking, 220 for SNP smoking, and 214 for SNP non-smoking groups.

In order to analyze P300 amplitude as the defining phenotype and as a continuous trait, model-free sib-pair quantitative trait linkage (QTL) analysis was performed using SIBPAL. Evidence for linkage was found on chromosome 7 using both MS markers (asymptotic *p *= 6.9 × 10^-6 ^and empirical *p *= 5.0 × 10^-5 ^at 158 cM; Figure 3A) and SNPs (asymptotic *p *= 2.3 × 10^-5 ^and empirical *p *= 1.4 × 10^-4 ^at 176 cM; Figure 3B). We also evaluated the effect of smoking on the linkage signal provided by P300 amplitude. With the SNP genome scan, the non-smokers provided evidence for linkage (asymptotic *p *= 2.8 × 10^-5 ^and empirical *p *= 3.7 × 10^-4 ^at 166 cM; Figure 3B), while the smokers did not. A similar trend was seen with the MS genome scan (Figure 3A).

**Figure 4:**
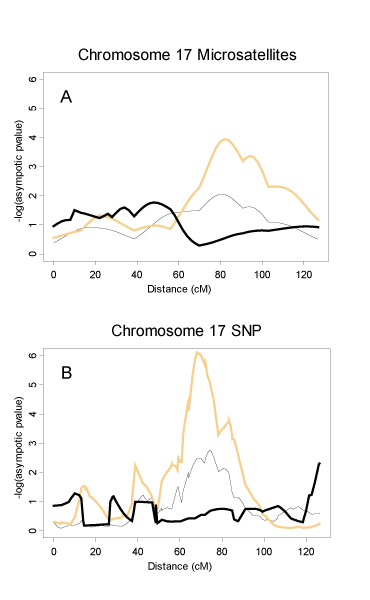
**Sib-pair QTL results of P300 amplitude, stratified for alcoholism**. Linkage results are shown for MS markers (A, *N *= 735 sib-pairs) and SNPs (B, *N *= 718 sib-pairs). The negative log of the asymptotic *p*-value is plotted over distance in centimorgans (cM). Results are shown for the entire group (thin black line), the alcoholism subgroup (thick tan line), and the non-alcoholism subgroup (thick black line). Sample sizes in sib-pairs are 173 for MS alcoholism, 104 for MS non-alcoholism, 170 for SNP alcoholism, and 103 for SNP non-alcoholism subgroups.

Repeating the P300 analysis stratifying sib pairs into alcoholic and non-alcoholic subgroups showed linkage to chromosome 17 in the alcoholism subgroup using SNPs (asymptotic *p *= 7.64 × 10^-7^; empirical *p *= 9.0 × 10^-5 ^at 68 cM; Figure 4B) and to a lesser extent using MS markers (asymptotic *p *= 0.0001; empirical *p *= 0.001 at 80 cM; Figure 4A). This signal on chromosome 17 was not seen in the group as a whole or in the non-alcoholism subgroup.

The inclusion of alcoholism as a covariate had little impact on the P300 signal on 7q, and similarly smoking status had no impact on the P300 signal on 17q. No evidence for linkage was seen on chromosome 12q with the QTL analyses. Restricting the analyses to only those relative pairs that had both MS and SNP genome scans had no significant effect on the results above.

## Discussion

ARP linkage analysis using SNP marker data suggests a susceptibility locus for alcoholism on chromosome 10q. Including P300 amplitude as a covariate in the analysis provides additional evidence of linkage to chromosome 12q in those ARPs with low P300 amplitude. QTL analysis of P300 amplitude shows significant evidence of linkage to chromosome 7q among non-smokers. QTL analysis of P300 amplitude also shows evidence for linkage to chromosome 17 among alcoholics. Our results suggest separate genetic determinants for the effects of alcoholism and smoking status on P300 amplitude, with linkage to chromosomes 12 and 17 in alcoholic individuals, and linkage to chromosome 7 in non-smokers. The 12q locus appears to be associated with alcoholism and low P300 amplitude, while the 17q locus appears to be associated with the variability in P300 in alcoholics, regardless of the absolute amplitude.

Differences between SNP and MS data could be due to genotyping errors, errors in marker order or intermarker distances, differences in informativeness of SNP vs. MS markers, and the presence of linkage disequilibrium. Initially, we anticipated that the SNP genome scan would provide greater power to detect linkage and/or greater precision in localizing the linkage signal compared to the MS genome scan, due to the presumably higher marker information content and greater density of markers in the SNP genome scan. However, the results were remarkably similar. One possible explanation for the similarity in the results is that the study was already adequately powered for a MS genome scan, and the SNP genome scan provided little additional power. One would expect SNP genome scans to have a greater impact for studies with small sample size.

The region on chromosome 12, detected in alcoholics with low P300 amplitude, lies in the vicinity of the mitochondrial aldehyde dehydrogenase gene (ALDH2), long implicated to have a role in alcohol dependence. Interestingly, linkage analysis with a parent-of-origin model also detects linkage to this same region on chromosome 12, suggesting the presence of an imprinted gene in this region influencing susceptibility to alcoholism.

## Conclusion

In this study the use of covariate-based linkage analysis and the inclusion of parent-of-origin effects identified possible linkage signals that would not have been detected otherwise, suggesting an important role of such strategies in the dissection of genetic heterogeneity in complex disease.

## Abbreviations

ARP: Affected relative pair

COGA: Collaborative Study on the Genetics of Alcoholism

IBD: Identity-by-descent

MS: Microsatellite

QTL: Quantitative trait linkage

SNP: Single-nucleotide polymorphism

## Authors' contributions

JFB helped design the study, performed affected relative-pair and parent-of-origin linkage analyses, and drafted the manuscript. SREQ performed sib-pair linkage analyses and drafted the manuscript. ARP performed IBD calculations. KABG designed and coordinated the study and helped draft the manuscript. All authors read and approved the final manuscript.
